# Are There Abnormalities in Peripheral and Central Components of Somatosensory Evoked Potentials in Non-Specific Chronic Low Back Pain?

**DOI:** 10.3389/fnhum.2016.00521

**Published:** 2016-10-17

**Authors:** Christian Puta, Marcel Franz, Kathrin R. Blume, Holger H. W. Gabriel, Wolfgang H. R. Miltner, Thomas Weiss

**Affiliations:** ^1^Department of Sports Medicine and Health Promotion, Friedrich Schiller University JenaJena, Germany; ^2^Center for Interdisciplinary Prevention of Diseases Related to Professional Activities, Friedrich Schiller University JenaJena, Germany; ^3^Department of Biological and Clinical Psychology, Friedrich Schiller University JenaJena, Germany

**Keywords:** somatosensory evoked potential (SEP), median nerve stimulation, N9 SEP component, Erb’s point, N20 SEP component, non-specific chronic low back pain (CLBP)

## Abstract

Chronic low back pain (CLBP) was shown to be associated with longer reflex response latencies of trunk muscles during external upper limb perturbations. One theoretical, but rarely investigated possibility for longer reflex latencies might be related to modulated somatosensory information processing. Therefore, the present study investigated somatosensory evoked potentials (SEPs) to median nerve stimulation in CLBP patients and healthy controls (HC). Latencies of the peripheral N9 SEP component were used as the primary outcome. In addition, latencies and amplitudes of the central N20 SEP component, sensory thresholds, motor thresholds and nerve conduction velocity were also analyzed in CLBP patients and HC. There is a trend for the CLBP patients to exhibit longer N9 latencies at the ipsilateral Erb’s point compared to HC. This trend is substantiated by significantly longer N9 latencies in CLBP patients compared to normative data. None of the other parameters showed any significant difference between CLBP patients and HC. Overall, our data indicate small differences of the peripheral N9 SEP component; however, these differences cannot explain the reflex delay observed in CLBP patients. While it was important to rule out the contribution of early somatosensory processing and to elucidate its contribution to the delayed reflex responses in CLBP patients, further research is needed to find the primary source(s) of time-delayed reflexes in CLBP.

## Introduction

Back pain has become one of the biggest problems of public health in the Western countries. Low back pain has a lifetime prevalence of 84%. The prevalence of chronic low back pain (CLBP) is 23%, and 12% of the population in the Western countries is disabled by low back pain (Airaksinen et al., 2006). A specific structural pathology cannot be identified in about 85% of patients (Deyo and Weinstein, 2001); therefore, this pain syndrome is commonly classified as non-specific CLBP. Previous studies have demonstrated several changes of the sensorimotor system in CLBP patients. Among others, CLBP patients show impairments of muscle relaxation (Paquet et al., 1994), postural control (Radebold et al., 2000), proprioception (Brumagne et al., 2004), respond with pain to nociceptive stimuli of low intensity (Puta et al., 2012, 2013), and show cortical reorganization of representation areas of the back compared to healthy controls (HC; Flor et al., 1997). Data also indicate that CLBP subjects show significant changes of body image and a decrease of tactile acuity in the body area affected by pain (Moseley, 2008).

With respect to motor control, several studies demonstrated altered activation of trunk muscles (for review see van Dieën et al., 2003). While some studies indicated delayed activation of deep abdominal muscles (e.g., Hodges and Richardson, 1996), others found changes in superficial trunk muscle activation during external perturbations in low back pain (Magnusson et al., 1996; Wilder et al., 1996; Radebold et al., 2000; Stokes et al., 2000; van Dieën et al., 2003). Furthermore, trunk muscles demonstrated delayed reflex responses after external perturbations (Reeves et al., 2005; Liebetrau et al., 2013; Navalgund et al., 2013). Consistent with these findings, we showed longer reflex response latencies (~15 ms delay; range from 6 ms to 21 ms) of CLBP patients’ trunk muscles to unpredictable external upper limb perturbations during upright standing (Liebetrau et al., 2013). Our model-based approach indicated that such reflex delays of superficial abdominal muscles might impair spinal stability (Liebetrau et al., 2013). These experimental and model-based results contribute to the on-going discussion (Reeves et al., 2009; Wulf et al., 2012) about the clinical relevance and the possible underlying mechanism of the longer reflex latencies in non-specific CLBP.

The time delayed reflex responses in CLBP might be due to several processes: altered thresholds of somatosensory activation, lowered conduction velocity of somatosensory nerves, changes of excitability or inhibition of spinal cord structures, changes of supraspinal spinal cord excitability, lowered conduction velocity of motor nerves, altered thresholds of muscle activation, or a combination of these factors. Some studies demonstrated changes at the peripheral level in chronic non-specific and pseudoradicular back pain. Freynhagen et al. (2008) found subclinical sensory impairments at the affected foot dorsum as indexed by increased mechanical and vibratory detection thresholds using quantitative sensory testing (QST) in patients with pseudoradicular back pain. Moreover, a hypersensitivity to pressure pain and a hyposensitivity to vibration were found at the affected region in female back pain patients at the threshold level (Blumenstiel et al., 2011). Furthermore, we found “pinprick allodynia” (Puta et al., 2012) to punctate low-intensity stimuli, and somatosensory abnormalities for painful and innocuous stimuli in female non-specific CLBP patients (Puta et al., 2013). Importantly, these somatosensory abnormalities were detected both at the affected body site (dorsum of the lower back) and at a site distinct from the region of pain, the dominant hand. Both abnormalities might contribute to the increased reflex latencies described above. In addition, reduced conduction velocities might lead to a time delay in the reflex responses. Moreover, a descriptive study in patients with specific low back pain (lumbar spinal stenosis) identified some patients with altered somatosensory evoked potentials (SEPs) *and* motor evoked potentials (Leinonen et al., 2002). For example, latency of the N40 component was prolonged in one of 26 patients and a lack of responses was observed in two other patients. In addition, smaller magnitude of the N40 component was observed in 61% of patients with lumbar spinal stenosis. Nevertheless, it remains unclear whether early somatosensory processing in CLBP patients is altered.

Therefore, the present study aims to investigate latencies of the N9 SEP component following electrical stimulation of the median nerve in CLBP patients and HC as primary outcome. In addition, latencies and amplitudes of the N20 SEP component, sensory thresholds, motor thresholds and nerve conduction velocity for stimulation of the median nerve were assessed. Median nerve was used because altered reflexes responses were found for trunk muscles to perturbations initiated via the hand (Leinonen et al., 2001, 2007), and somatosensory abnormalities at threshold level were identified at the hand (Puta et al., 2012, 2013) in CLBP.

## Materials and Methods

### Subjects

Eleven subjects with CLBP (mean age: 39.2 years; age range: 22–63 years, 7 females) and 10 age- and gender matched HC (mean age: 37.2 years; age range: 24–62 years) participated in this study. CLBP patients met the following inclusion criteria: (1) minimum of 3 months history of low back pain; (2) pain has been classified as “non-specific low back pain” (no indicators for nerve root problems, e.g., unilateral leg pain, radiating to foot or toes, numbness and/or paresthesia); (3) no psychiatric disorders, no disease associated with small fiber pathology (e.g., diabetes mellitus) according to medical history; and (4) no other chronic disorder. None of the HC reported lasting pain episodes (>1 month), current pain, or any neurological, psychiatric or other chronic disease. The study was carried out in accordance with the Declaration of Helsinki and was approved by the Ethics committee of the Friedrich Schiller University Jena (3039-02/11). All subjects gave written informed consent after having been thoroughly informed about the nature and course of the experiment.

### Characterization of CLBP Patients and HC

Table 1 shows typical characteristics of CLBP patients and HC subjects. Groups were matched with respect to sex and age. Table 1 shows clear differences of pain parameters between groups. Back pain intensity was assessed using a visual analog scale (0 = “no pain” and 10 = “most intense pain imaginable”) for the actual pain (VAS^actual^) and the average pain during the last 4 weeks (VAS^4 weeks^; pain intensity rating in response: “How would you rate your *average* pain over the last 4 weeks?”). Pain*DETECT* questionnaire (© Pfizer Pharma GmbH 2005, Pfizer bv 2009. Cappelle a/d IJssel, Netherlands) screens the presence of neuropathic pain without physical examination (Freynhagen et al., 2006). The following cut off values are found to be most appropriate (Freynhagen et al., 2006): “negative”, a neuropathic pain component is unlikely (<15% score range 0–12); “unclear” result is ambiguous, a neuropathic pain component can be present (score range 13–18); or “positive”, a neuropathic component is likely (>90%, score range 19–38).

**Table 1:** **Characteristics of the chronic low back pain (CLBP) patients and healthy controls (HC) and the somatosensory evoked potential (SEP) parameters to the median nerve stimulation**.

No.	Age (Years)	Height (cm)	Weight (kg)	Sex	Pain duration	VAS^actual^	VAS^4weeks^	Pain detect	BDI	N9-L (ms)	N9-A (mV)	N20-L (ms)	N20-A (mV)	S. Thr. (a.u.)	M. Thr. (a.u.)	NCV (m/s)
CLBP1	31	183	74	m	>24	0.0	5.0	5^a^	8	11.00	−9.398	20.20	−3.472	−	540	60.00
CLBP2	54	164	57	f	>24	0.7	2.0	1^a^	6	9.60	−6.653	21.00	−2.653	110	220	60.42
CLBP3	30	183	68	m	24	1.8	3.0	5^a^	4	10.40	−3.140	19.40	−1.250	230	480	63.46
CLBP4	31	178	92	m	48	0.8	7.5	7^a^	11	10.40	−3.508	19.00	−1.933	250	750	62.02
CLBP5	64	163	80	f	480	3.1	5.2	5^a^	14	10.00	−0.314	18.40	−2.352	200	650	61.00
CLBP6	54	162	50	f	420	2.7	1.5	4^a^	4	10.60	−4.380	19.40	−2.337	250	650	52.83
CLBP7	58	168	57	f	240	4.2	4.9	6^a^	11	10.00	−3.809	18.80	−2.829	200	300	56.00
CLBP8	27	167	62	m	48	1.2	3.0	13^b^	3	9.80	−4.493	18.20	−1.506	150	900	59.69
CLBP9	36	170	80	f	162	7.6	5.0	23^c^	38	10.60	−1.991	19.00	−3.373	400	675	57.08
CLBP10	24	171	62	f	45	3.8	3.0	1^a^	5	10.60	−2.981	19.80	−0.601	200	500	58.49
CLBP11	22	174	63	f	48	1.3	2.2	1^a^	3	10.40	−5.142	19.60	−5.427	250	750	57.21
**Mean ± SD**	**39.2 ± 15.2**	**171 ± 8**	**67.7 ± 12.5**	**7f/4m**	**168 ± 175**	**2.5 ± 2.2**	**3.9 ± 1.8**	**6.5 ± 6.5**	**9.7 ± 10.1**	**10.3 ± 0.4**	**−4.2 ± 2.4**	**19.4 ± 0.8**	**−2.5 ± 1.3**	**224 ± 77**	**583 ± 201**	**58.9 ± 3.0**
HC1	25	182	85	m					3	10.60	−3.164	19.60	−1.770	220	570	60.38
HC2	54	155	55	f					0	9.40	−5.334	18.40	−0.757	200	500	56.91
HC3	28	181	80	m					2	9.80	−7.166	19.00	−3.420	200	650	61.73
HC4	25	180	68	m					4	10.4	−7.723	20.00	−3.815	600	1900	69.71
HC5	62	169	87	f					6	10.20	2.485	19.60	−1.522	200	1000	56.86
HC6	50	165	63	f					9	9.20	−4.461	19.40	−3.076	250	575	63.04
HC7	61	170	66	f					0	10.60	−1.454	20.40	−4.076	200	650	57.08
HC8	27	173	72	m					0	10.40	−4.412	19.80	−2.281	300	700	60.10
HC9	36	172	63	f					1	9.00	−9.347	18.80	−4.022	250	900	66.11
HC10	24	173	85	f					9	9.60	−3.416	19.20	−3.202	200	375	64.06
**Mean ± SD**	**37.2 ± 15.8**	**172 ± 8**	**72.4 ± 11.2**	**7f/4m**	**0.0 ± 0.0**	**0.0 ± 0.0**	**0.0 ± 0.0**	**0.0 ± 0.0**	**3.4 ± 3.5**	**9.9 ± 0.6**	**−4.4 ± 3.4**	**19.4 ± 0.5**	**−2.8 ± 1.2**	**262 ± 123**	**782 ± 433**	**61.6 ± 4.3**
**CLBP vs. HC**	***P* = 1.0^§^**	***P* = 0.80^§^**	***P* = 0.38^§^**		**−**	***P* < 0.01^§^**	***P* < 0.001^§^**	**−**	^**#**^***P* = 0.024**	^**§**^***P* = 0.096**	^**§**^ ***P* = 0.86**	^**§**^ ***P* = 0.81**	^**§**^***P* = 0.62**	^**#**^***P* = 0.63**	^**#**^***P* = 0.39**	^**§**^***P* = 0.11**
									**(0.82^*d*^)**	**(0.76^*d*^)**	**(0.08^*d*^)**	**(0.04^*d*^)**	**(0.22^*d*^)**	**(0.37^*d*^)**	**(0.59^*d*^)**	**(0.73^*d*^)**

*VAS^actual^, current pain intensity before testing on visual analog scale (0 = “no pain” and 10 = “most intense pain imaginable”); VAS^4weeks^ pain intensity rating in response: “How would you rate your average pain over the last 4 weeks?”; All participants were employed. a.u., arbitrary units; m, male; f, female; PainDETECT; classification as nociceptive^a^. unclear^b^. neuropathic^c^ pain. BDI, Beck Depression Inventory; N9 and N20 (N9 and N20 SEP component). L, latency; A, amplitude. S.Thr. and M. Thr, somatosensory (S) and motor (M) threshold (Thr); NCV, nerve conduction velocity. ^§^t-test for unpaired samples; ^#^Mann-Whitney-U-Test; ^d^Effect size Cohens d*.

### SEP Recording and Data Processing

**Figure 1:**
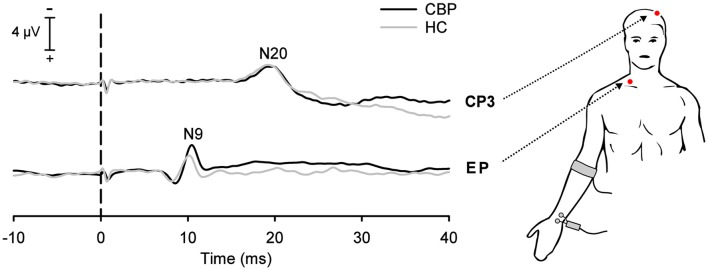
**Grand average somatosensory-evoked potentials (SEPs) in chronic low back pain (CLBP) patients and healthy controls (HC) following stimulation of the median-nerve of the right upper extremity.** Negative SEP components were recorded at the ipsilateral Erb’s point (EP, N9 component) and at the contralateral centroparietal scalp site (CP3, N20 component). Vertical dashed line indicates the electrical stimulus onset.

Subjects were lying on bench in a quiet, darkened and electrically shielded room kept at 21–23°C. Participants were instructed to keep their eyes closed and to relax during all recordings. SEPs were recorded in response to stimulation of the median nerve of the right arm. Stimulation was carried out using a bipolar pad electrode (inter-electrode distance: 2 cm; Technomed Europe, Maastricht, Netherlands). The anode was placed on the wrist crease and distal to the cathode in order to avoid anodal block (Cruccu et al., 2008). A ground electrode was attached to the right arm between the stimulation site and the recording electrodes to reduce stimulus artifacts. Recording electrodes were placed at two locations: (1) Erb’s point (2–3 cm above the clavicle) with the active electrode ipsilateral to the stimulation (EPi) referenced to the contralateral Erb’s point (EPc); and (2) a contralateral scalp electrode (centroparietal, CP3) referenced to a frontal scalp electrode (Fz). The recordings at Erb’s point (EPi-EPc) captures the N9 component of SEP and indicates the arrival of the afferent nerve volley at the brachial plexus (see Figure 1), while the recordings at CP3 characterizes the early N20 component of the cortical SEP. Electrode impedances were kept below 5 kΩ throughout the session. Electrical stimuli consisted of a train of biphasic constant current square wave pulses (0.2 ms duration per pulse; DS5, Digitimer, Hertfordshire, UK) delivered at a constant frequency of 2.5 Hz. Each recording session comprised 300 such trains. To obtain adequate SEP’s, the sum of the intensities for motor and sensory threshold was used to determine the individual stimulus intensity as indicated by a small reproducible muscle switch (Cruccu et al., 2008). Electrophysiological signals were amplified, analog band-pass filtered (0.1–1500 Hz), digitized at 5000 Hz (16 bit resolution), and stored to hard disk for offline analysis.

The distance between the stimulation electrode (cathode) and the Erb’s point was measured at the end of the experiment while the subject was standing with arms held horizontally. Conduction velocity was calculated as the quotient of this distance and the latency of the N9 component.

SEP data were processed using BrainVision Analyzer 2.0 (Brain Products, Gilching, Germany). Continuous recordings were digitally filtered (band-pass at 3–750 Hz; 12 dB/oct roll-off), segmented (−100 ms to 100 ms relative to electrical stimulus onset), and baseline corrected using the average electrical activity within the prestimulus interval from −20 ms to 10 ms. Finally, segmented epochs were averaged for each electrode site and each subject. Baseline-to-peak amplitudes and peak latencies of SEP components N9 and N20 were exported to SPSS for further statistical analysis.

### Data Analyses

All statistical calculations were carried out using IBM SPSS Statistics 21 (SPSS Inc., Chicago, IL, USA). SEP latencies and amplitudes of N9 and N20 components were tested for normal distribution using Shapiro-Wilks test. Our primary hypothesis was tested comparing N9 latencies of CLBP patients and HC by a *t*-test in accordance with the gatekeeping procedure of the IMMPACT-recommendations by Turk et al. (2008). For consistency, we used separate *t*-tests to analyze differences of N20 latency as well as the amplitudes of SEP components between CLBP patients and HC, respectively.

We also compared our data with respect to normative data for young adults (Mauguiére et al., 1999; Cruccu et al., 2008) to prove the quality of our data and to compare the CLBP findings with a much larger database of HC subjects. For this reason, our data of HC and CLBP subjects were *z*-transformed with respect to reference data (Cruccu et al., 2008). *Z*-scores for all subjects of both groups were transformed with respect to reference data using the following expression (Magerl et al., 2010):

(1) $$\begin{array}{l} \begin{array}{lll} z-\text{score}\; & =\;(\text{single}\;\text{subject}-{\text{mean}}_{\text{control}\;\text{from}\;\text{normative}\;\text{data}})/ & \end{array}\\ \;{\text{SD}}_{\text{control}\;\text{from}\;\text{normative}\;\text{data}}. \end{array}$$

Then, each CLBP patient’s data was compared with the group mean of normative data with the 95% confidence interval (CI) of a standard normal distribution defined as follows (Rolke et al., 2006): 95% CI = Mean _normative data_ ± 1.96 SD _normative data_.

The significance of differences for the N9 component was estimated comparing patients’ mean ± SD obtained by *z*-normalization vs. a standard normal distribution (i.e., mean ± SD = 0 ± 1) of an equal number of HC using the internet-based statistical freeware Simple Interactive Statistical Analysis (SISA^1^; Magerl et al., 2010; Maier et al., 2010; Pellkofer et al., 2013).

Finally, separate *t*-tests for unpaired samples were used to compare somatosensory thresholds, motor thresholds, nerve conduction velocity, pain intensity ratings (VAS values), pain*DETECT* values, and Beck Depression inventory (BDI) values between CLBP patients and HC.

## Results

### Demographic and Clinical Characteristics of the CLBP Patients and Healthy Controls

Demographic data and clinical characteristics of CLBP patients and HC are presented in Table 1. Among the CLBP patients, pain*DETECT* classified nine patients as “negative” (a neuropathic pain component is unlikely), one CLBP patient was classified as “positive” (a neuropathic pain component is likely), and one CLBP patient was categorized as “unclear” (Table 1). Depression was assessed using a German version (Hautzinger et al., 1994) of the BDI (Beck et al., 1961). Seven CLBP patients showed no clinically relevant BDI scores (<10), three CLPB patients reported light depressive symptoms (score value: 10–17), and one patient received a BDI score of 38 (Table 1). Ten of the healthy subjects reported no clinically relevant BDI score (<10), one showed a BDI score of 19. We excluded this subject from all further analyses. All CLBP patients and all HC reported to be without any analgesic medication for at least 48 h before examination.

### Somatosensory Evoked Potentials (SEP)

#### Primary Outcome: Latencies of the N9 Component of the SEP

The N9 component of the SEP recorded at Erb’s point (N9) showed a trend to have a longer latency (0.4 ms; Figure 1) in CLBP patients as compared to HC (*T* = −1.75, *P* = 0.096; see Table 1).

**Figure 2:**
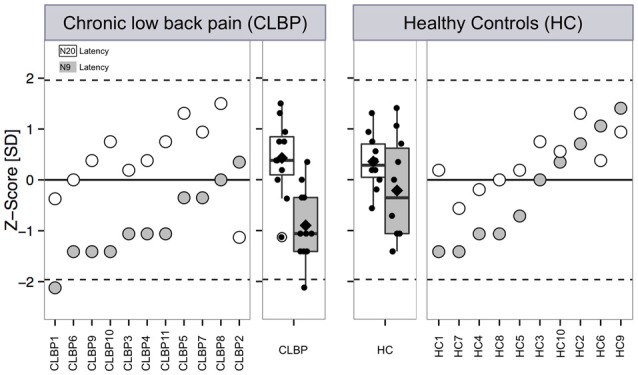
**Latencies of the N9 and N20 components after electrical stimulation at the median nerve of CLBP patients and age-/gender-matched HC.** Latencies of the N9 and N20 SEP components are presented as single subject data and boxplots expressed as *z*-scores with respect to reference data are based on typical latency values of SEPs of young adults (Mauguiére et al., 1999; Cruccu et al., 2008). *Z*-score values above “0” indicate a gain of function when subject SEP latency is shortened compared to the reference data, while *z*-scores below “0” indicate a loss of function when the SEP latency is prolonged compared to reference data. Filled symbols and boxes: latency of the N9 SEP component. White symbols and boxes: Latency of the N2 SEP component. Boxplots present median (black line), mean (rectangle), first (Q1) and third quartile (Q3) and Q1–1.5 × interquartile range and Q3 + 1.5 × interquartile range. CLBP, chronic back pain patients; HC, healthy controls.

Figure 2 outlines our findings with respect to normative data for young adults (Mauguiére et al., 1999; Cruccu et al., 2008). All HC subjects and all but one CLBP patients were within the normative range of latencies (CLBP 1 in Table 1). This single subject showed a *z*-value of less than −2 for the N9 component (resulting from the original latency of 11.0 ms; Figure 2 left panel, filled circle). Assessment of the differences between CLBP patients and HC for N9 latencies using *z*-normalization of an equal number of HC (Magerl et al., 2010) confirmed the significantly longer latencies in CLBP patients (*T* = 2.411; *P* = 0.028).

#### Amplitude of the N9 Component of the SEP

Comparing the amplitudes for the N9 SEP component between CLBP patients and HC, *t*-test revealed no significant differences for the N9 amplitude (*T* = −0.19, *P* = 0.855).

#### Latencies of the N20 Component of the SEP

No significant differences between CLBP patients and HC were found for N20 latencies (*T* = 0.239, *P* = 0.813; Figure 1). All HC subjects and all CLBP patients were within the normative range (Figure 2; Table 1). Also, no significant difference of N20 latencies were observed for CLBP patients in comparison to normative data (*T* = −1.135, *P* = 0.279; Figure 2).

#### Amplitudes of the N20 Components of the SEP

*T*-test revealed no significant differences for the N20 amplitudes (*T* = −0.507, *P* = 0.618) between CLBP patients and HC.

### Sensory Thresholds, Motor Thresholds and Nerve Conduction Velocity

*T*-tests for independent samples revealed no significant difference between CLBP patients and HC for sensory thresholds to electrical stimulation of the median nerve (*T* = 0.827, *P* = 0.419). Similarly, no significant differences between CLBP patients and HC were found for motor thresholds to electrical stimulation of the median nerve (*T* = 1.327, *P* = 0.186). Finally, conduction velocities (see “Materials and Methods” Section) were not significant between groups (*T* = 1.673, *P* = 0.111).

## Discussion

The primary aim of the present study was to investigate N9 latency of SEPs in response to median nerve stimulation in CLBP patients and HC to assess whether the timing of this component might explain the longer reflex latencies observed in CLBP patients (Liebetrau et al., 2013). CLBP patients exhibited a trend to longer peripheral N9 latencies compared to sex- and age-matched HC. There were no significant changes in N9 amplitude, N20 latency, amplitude, or sensory and motor thresholds to median nerve stimulation.

### Primary Aim: N9 Component

Our results demonstrate for the first time that there is only a small temporal difference of 0.4 ms in latency of the N9 component measured at Erb’s point between CLBP patients and HC. This small delay in CLBP can hardly account for the time delayed paraspinal or abdominal reflex responses observed in these patients (e.g., Liebetrau et al., 2013; Navalgund et al., 2013); at maximum, they contribute minimally to the time delayed reflex responses in CLBP patients. The range of delayed reflex responses in CLBP patients was 5.4 ms for the paraspinal muscles (Navalgund et al., 2013) and 21 ms for the abdominal muscles (Liebetrau et al., 2013). So the delay of the SEP latencies for the N9 component between CLBP patients and HC only explains 7.4% of the variance of the time delayed reflexes in CLBP patients. Such a small difference is in line with results by Rossi et al. (2003) who demonstrated that experimentally induced tonic pain does not modify the peripheral sensory nerve action potential or the spinal processing of somatosensory stimuli.

### N20 Component

We found no differences in the N20 component neither for amplitude nor for latency in CLBP patients compared to HC. Thus, the minimal difference in latency observed in the N9 component has no influence on the primary central processing of somatosensory information, i.e., the primary somatosensory information processing of the upper extremity does not seem to be influenced by the back pain. This result is in contrast to Rossi et al. (2003) who demonstrated changes of N20 and later components in response to experimentally induced tonic pain. However, there seems to be an important difference between our study and the one by Rossi et al. (2003) study. Rossi et al. (2003) provoked tonic pain within the territory under investigation at the upper extremity, whereas we investigated the upper extremity in CLBP patients, i.e., in a non-affected region. Similarly, a study of laser-evoked potentials demonstrated no differences between CLBP patients and healthy subject also within the affected region (Franz et al., 2014) in the affected territory of the skin. Obviously, different submodalities of the somatosensory system seems to be influenced differentially by experimentally induced pain and chronic pain.

### Limitations and Future Directions

Our sample size with 21 subjects is relatively small. However, we observed a small difference in the N9 SEP latencies between CLBP patients and HC. It was important to rule out the contribution of early somatosensory processing and to elucidate its contribution to the delayed reflex responses in CLBP patients. Calculation of the two-sided 95% CI for the differences between the two groups for N9 SEP latency using G*power 3 (Faul et al., 2007) resulted in an CI of −0.1 ms to 0.9 ms. Therefore, the maximally explained variance of latency changes of the reflex responses of 5.4–21 ms in CLBP patients (Liebetrau et al., 2013) for the upper limit is less than 17%. We decided not to increase group sizes as the principal result of the study would not change, i.e., the peripheral N9 SEP latency changes seems to have only a small impact for the time delayed reflexes.

Recording of the N13 might have given additional information to the flow of somatosensory information. However, according to Stöhr et al. (2005), the mean amplitude of the N13 is more than half as small than the N9 amplitude. This would result in a 4-fold higher number of trials in order to receive the same signal-to-noise ratio as for the N9, which would prolong the experiment considerably. Moreover, first attempts to record the N13 were difficult as back pain patients had numerous (especially muscular) artifacts. Therefore, we decided not to include the N13 to this investigation. Finally, the results of N9 and N20 make it likely that this type of processing plays not an important role in the explanation of the reflex perturbations.

Our data indicate only small changes in early somatosensory information processing (0.4 ms delayed N9 SEP latency) and an unchanged amplitude of the N9 SEP component. Therefore, this early somatosensory processing can only account for a minor part of the delay reflex responses in CLBP patients. The reflex responses following sudden external perturbations applied to the trunk or via the upper limb are within the range of 40 ms and 120 ms (Radebold et al., 2001; Granata et al., 2004; Cholewicki et al., 2005; Rogers and Granata, 2006; Liebetrau et al., 2013), which correspond to M2 (medium latency) responses. The neural processing of M2 responses consists of a peripheral afferent component (early somatosensory information processing) plus neural processing within spinal and higher order circuits (Marsden et al., 1976; Nashner, 1976; Grey et al., 2001) plus the efferent pathway to the muscle. As the early somatosensory processing accounts only for a minor part of delay, the main proportion of the longer latencies of reflex responses in CLBP could be associated with the neural processing on spinal and higher order circuits and the efferent pathway to the muscle, as has been supposed by Nijs et al. (2012) pointing to a nociception-related alteration of the function of premotor interneurons in spinal proprioceptive pathways.

We used VAS for the characterization of our CLBP patients. While VAS is commonly used as the outcome in clinical and experimental studies also for CLBP (Mannion et al., 2007), there are questions regarding the use of VAS in research studies. Bodian et al. (2001) characterized three important open issues: optimal connection between VAS values and clinical experience outside research settings, the question of whether earlier values should be visible or not, and the question whether VAS measurement itself influences on subjective feelings of patients and outcome parameters. Different pain measures have been compared showing that binary formats are preferred by patients (Rothaug et al., 2013). Nevertheless, there is no single best measure for the pain assessment in CLBP (Mannion et al., 2007). We used the VAS due to its simplicity, reliability, validity and its ratio scale properties (Bodian et al., 2001).

## Conclusion

Investigating CLBP patients and sex- and age-paralleled HC, we found a small difference in the latency of the N9 component of the SEP between groups. The latency was 0.4 (95% CI: −0.1 to 0.9) ms longer in CLBP patients. This small difference in N9 latency together with our additional results (e.g., the unchanged N20 latency) indicate that changes in peripheral somatosensory processing are not able to explain the time delay of 5–21 ms of trunk muscles in CLBP patients after sudden unexpected external perturbations as reported by several groups.

## Author Contributions

CP, TW and HHWG: designed the experiment. MF and KRB: gathered data. CP and MF: conducted data analysis. CP, MF, TW and WHRM: wrote the manuscript. All authors discussed the results and its implications, commented and edited the manuscript at all stages, and approved the final version.

## Funding

This research was funded by the German Federal Ministry of Education and Research, BMBF grant (No. 01EC1003B).

## Conflict of Interest Statement

The authors declare that the research was conducted in the absence of any commercial or financial relationships that could be construed as a potential conflict of interest.

## Footnotes

^1^http://www.quantitativeskills.com/sisa/
